# Reverse causation between multiple sclerosis and psoriasis: a genetic correlation and Mendelian randomization study

**DOI:** 10.1038/s41598-024-58182-9

**Published:** 2024-04-17

**Authors:** Hao Zhou, Yajie Qi, Yingxin Xu, Xiaoyi Qi, Hui Qi

**Affiliations:** 1 Peking University Shenzhen Hospital Clinical College, Anhui Medical University, Shenzhen, 518036 China; 2 The Fifth Clinical Medical College, Anhui Medical University, Hefei, 230000 China; 3 Peking University Shenzhen Hospital, Shenzhen, 518036 China; 4 Medical College, Shantou University, Shantou, 515000 China

**Keywords:** Genetics, Neuroscience, Medical research, Neurology, Rheumatology, Risk factors

## Abstract

Observational studies have found a potential bidirectional positive association between multiple sclerosis and psoriasis, but these studies are susceptible to confounding factors. We examined the directionality of causation using Mendelian randomization and estimated the genetic correlation using the linkage disequilibrium score. We performed Mendelian randomization analysis using large-scale genome-wide association studies datasets from the International Multiple Sclerosis Genetics Consortium (IMSGC, 115,803 individuals of European ancestry) and FinnGen (252,323 individuals of European ancestry). We selected several Mendelian randomization methods including causal analysis using summary effect (CAUSE), inverse variance-weighted (IVW), and pleiotropy-robust methods. According to CAUSE and IVW the genetic liability to MS reduces the risk of psoriasis (CAUSE odds ratio [OR] 0.93, *p* = 0.045; IVW OR 0.93, *p* = 2.51 × 10^–20^), and vice versa (CAUSE OR 0.72, *p* = 0.001; IVW OR 0.71, *p* = 4.80 × 10^–26^). Pleiotropy-robust methods show the same results, with all *p*-values < 0.05. The linkage disequilibrium score showed no genetic correlation between psoriasis and MS (rg =  − 0.071, *p* = 0.2852). In summary, there is genetic evidence that MS reduces the risk of psoriasis, and vice versa.

## Introduction

Multiple sclerosis (MS) is a demyelinating disease of the central nervous system that involves abnormal immune response. Around 2 million people throughout the world are affected by MS^1^. The disease causes the most neurological disability in young adults^2^. However, the precise immunological mechanism of MS is not completely understood. There is much evidence supporting the immunological aspect of MS, such as the presence of pathological markers of immune activity in central nervous system-related lesions^3^.

Psoriasis is a chronic relapsing skin disease. A recent epidemiological survey has shown that there are about 60 million cases of psoriasis worldwide^4^. There is growing research to suggest that psoriasis has systemic effects, meaning that it can affect organs beyond the skin, such as the cardiovascular system, joints, and central nervous system^5^. Similar to MS, psoriasis is a systemic inflammatory disease with considerable comorbidities^6–8^. Both diseases are likely mediated by cellular immunity^9,10^, and elevated levels of TNF-α and lL-17 are found both in psoriasis and in MS patients^11–13^. Since the two diseases share the same immune disorder mechanism, fumarates have become an effective drug in the clinical treatment of psoriasis and MS^14^.

Although the two diseases share the same immune disorder mechanism and the same drug regimen, it is not clear whether MS and psoriasis can be considered comorbidities. A number of previous observational studies and meta-analyses have revealed an increased risk of psoriasis in MS patients and vice versa^15–20^. However, it remains unclear whether there is a causative association between the two inflammatory diseases due to possible biases, including reverse causation and confounding factors.

MS and psoriasis share a number of risk factors, including smoking, obesity, and autoimmune diseases^21–25^, which might function as confounders that cause erroneous correlation and are challenging to eliminate in observational studies. Particularly, it has recently been discovered that smoking and obesity are causally linked to psoriasis^26,27^.

Mendelian randomization (MR) is an alternative approach to control the influence of observational bias^28,29^. MR relies on the concept that different genetic variations are randomly mixed during the process of meiosis, resulting in random distribution of genetic variants among individuals. It is determined at birth whether an individual inherits a certain genetic variant that influences risk factors or disease susceptibility. To some extent, the process by which an individual’s genetic code is generated resembles the concept of randomized clinical trial (RCT). Similar to how patients are randomly assigned to either a treatment group or a control group in RCT, genetic variations serve as “instruments” in a method called “instrumental variable” (IV) in MR analysis. Due to the fact that genetic variants are usually unrelated to confounders, any observed discrepancies in outcome between people who possess and those who do not possess the variant can be traced back to underlying discrepancies in risk factors or disease susceptibility. Therefore, MR provides a more credible comprehension of the impact of variable exposures on the feature of interest, in comparison with conventional observational studies, which might be affected by reverse causality and confounding^30^. During the MR analysis, we utilized the genetic variation strongly linked to exposure as IVs to investigate any causal relationship that might exist between the relevant exposure and the outcome^29^. Confounding and reverse causality biases do not affect MR estimations because genetic variants are assigned at random during conception. The most common causal inferences are based on single nucleotide polymorphisms (SNPs) selected as IVs in genome-wide association studies (GWAS)^29^. We carried out a bidirectional MR study to examine the causal association between MS and psoriasis, and a set of sensitivity analyses to take into consideration pleiotropic SNPs related to relevant confounding factors.

## Materials and methods

### Data sources for exposure and outcome

We conducted a bidirectional MR study through the use of summary statistics from public GWAS on psoriasis and MS including 5, 621 psoriasis cases and 252, 323 controls based on the FinnGen databases (Release 8, https://r5.finngen.fi/), as well as 47, 429 MS cases and 68, 374 controls from the International Multiple Sclerosis Genetics Consortium (IMSGC) study^31^. To reduce the possibility of bias in MR analyses due to population stratification, the summary statistics for both psoriasis and MS datasets were collected from persons of European ancestry. Ethical clearance was waived for the current study because the study only used the summary statistics from publicly available GWAS and made no attempt to recognize individual data. In the original studies, the ethics committees provided relevant approvals, and signed informed consent forms were available for all participants.

### MR assumptions and IV selection

**Figure 1:**
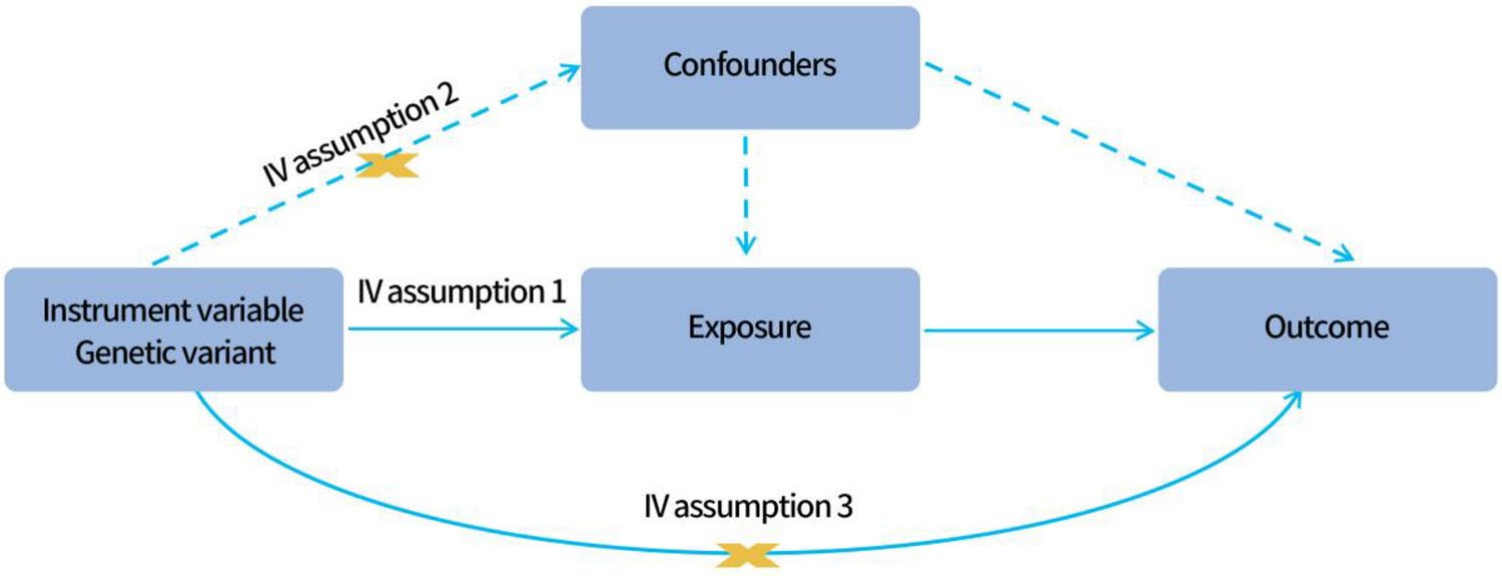
3 assumptions necessary for Mendelian randomization. Assumption 1: there should be a strong association between the instrument and the exposure. Assumption 2: there is no association between genetic variation and potentially confounding factors that are related with exposure and outcome. Assumption 3: genetic variation does not directly affect outcome unless it is affected by exposure.

For genetic variation to be usable as IVs in MR analysis, the following three critical assumptions must be true (Fig. 1): First, there should be a strong connection between the instrument and the exposure (correlation assumption). Second, genetic variation does not directly affect outcome unless it is affected by exposure, i.e. pleiotropy should not be present (exclusion limitation assumption). Third, there is no association between genetic variation and potentially confounding factors that are related to exposure and outcome (independence assumption)^32^.

IVs were selected using the following criteria. Initially, SNPs associated with exposure were selected with a significance threshold of *p* < 5 × 10^–8^ for psoriasis and MS. Subsequently, we computed pair-wise linkage disequilibrium and weeding out SNPs with r^2^ ≥ 0.01 in the specific genomic region (kb = 10,000). Third, the effects of genetic variations from different studies were coordinated and palindromic SNPs (i.e. SNPs in which the allele comprised self-complementary base sequences) were omitted. Fourth, when the outcome was affected by instruments in a way that was unrelated to the exposure, this was considered horizontal pleiotropy, which refuted the exclusion restriction and independence assumption. To minimize pleiotropy, we reviewed the known links between instruments and widespread risk factors, particularly alcohol consumption, obesity, and smoking in the GWAS Catalog (www.ebi.ac.uk/gwas/) and PhenoScanner (www.phenoscanner.medschl.cam.ac.uk/). Finally, as weak instrument (i.e., SNPs with low correlation to exposure) can decrease the rationality of the MR’s correlation assumption, we used the *F*-statistic to assess the strength of the instrument^33^. We did not make use of proxies to stand in for absent SNPs.

### Statistical analysis

We used an approach known as Causal Analysis Using Summary Effect Estimates (CAUSE)^34^ as our primary way of analysis since it has been shown to be more effective than other methods designed to identify causal linkages when pleiotropy is present^35^. In addition, we carried out a battery of sensitivity analyses, which incorporated pleiotropy-robust methods.

To begin, we aligned effect sizes and prevented strand mismatch by harmonizing the results of the summary statistics. Next, we performed CAUSE, which combined all genetic variations after clumping and consequently boosted statistical power, using MS as an exposure and psoriasis as an outcome, and vice versa. GWAS were utilized in CAUSE to differentiate the effects of causation (i.e., SNPs influence psoriasis via affecting MS) from correlated pleiotropy (i.e., SNPs are related to MS and psoriasis by a common heritable component, which contradicts the MR independence assumption), while accounting for irrelevant horizontal pleiotropy (which violates the exclusion limitation assumption that SNPs are correlated with MS by specific mechanism). This approach utilizes Bayesian modelling to evaluate if the sharing model (the model in which the cause effect is fixed at zero) suits the data at least as well as the causal model (the model that permits cause effect at non-zero)^34^.

To complete the MR, we performed the inverse-variance weighted (IVW) analysis by making use of the multiplicative random effects model^28^, whose high-quality assessments of causality between MS and psoriasis relied on all three realized assumptions^32^. Furthermore, we tackled the pleiotropy problem by utilizing a variety of pleiotropy-robust methods (radial regression, robust adjusted profile score [RAPS], weighted median, MR Pleiotropy RESidual Sum and Outlier [MR-PRESSO])^36^. Finally, we did Cochran Q^37^, leave-one-SNP-out analysis, and the MR-Egger intercept^38^ to investigate the heterogeneity of the MR study.

Linkage disequilibrium score (LDSC) regression method regressed χ^2^ statistics based on SNPs to determine the heritability of a single trait and the co-heritability of two traits^39^. LDSC regression can identify whether there are confounding factors in MR analysis, so we utilized LDSC to calculate the heritability (h^2^) of MS or psoriasis as well as the co-heritability between MS and psoriasis through investigating the genetic correlation (rg)^39^.

Statistical significance was defined as *P* < 0.05. All MR studies were carried out using R software (version 4.0.1.) and a series of R packages “CAUSE”, “TwoSampleMR” and “MR-PRESSO”. Reporting follows the latest STROBE-MR statement^40^.

## Results

### Characteristics of the included SNPs

First, we extracted 64 and 29 SNPs that highly correlated with psoriasis and MS, respectively, from the GWAS summary statistics as the initial IVs. Afterward, three SNPs for MS (rs1112718, rs4676756, rs9275602) and two SNPs for psoriasis (rs12663590, rs2395471) that were associated with obesity through PhenoScanner and GWAS Catalog were removed. Finally, we carried out MR analysis with 61 and 27 SNPs associated with MS and psoriasis, respectively, as the final IVs (Supplementary Tables S1–S2). All of the F statistics were higher than the empirical criterion of 10^41^, which indicated that all SNPs satisfied the criteria for acceptable validity. Furthermore, the high F-statistics of the instruments provided evidence that the MR correlation assumption was fulfilled (Supplementary Tables S1–S2).

### Causal association between MS and psoriasis

**Figure 2:**
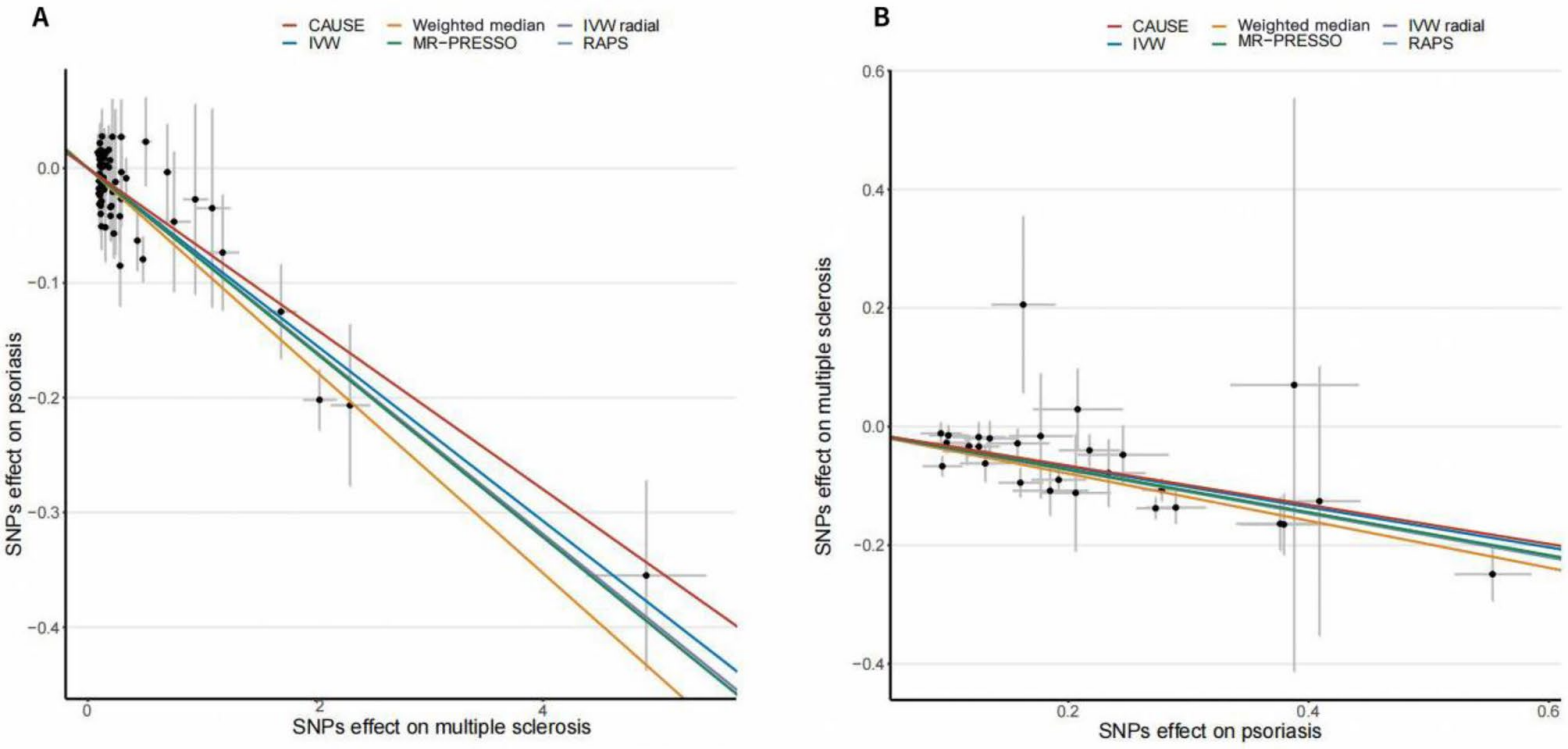
Plots of Mendelian randomization estimates of the causal relationship between multiple sclerosis and psoriasis. (**A**) The scattered plot of SNPs associated with multiple sclerosis and their risk on psoriasis. (**B**) The scattered plot of SNPs associated with psoriasis and their risk on multiple sclerosis. SNPs, single nucleotide polymorphisms.

CAUSE analysis indicated that the genetic liability to MS reduced the risk of psoriasis (odds ratio [OR] = 0.93, 95% credible interval [CredIn] = 0.87–1.00, *p* = 0.045). Similarly, CAUSE showed that the genetic liability to psoriasis reduced the risk of MS (OR 0.74, 95% CredIn = 0.54–1.02, *p* = 0.006) (Table 1, Fig. 2). The IVW approach yielded the same type of negative correlation effect (Table 1, Fig. 2). Pleiotropy-robust methods confirmed our research conclusions that the genetic liability to MS reduced the risk of psoriasis (1.76 × 10^–20^ ≤ *p* ≤ 1.52 × 10^–13^), and vice versa (4.96 × 10^–33^ ≤ *p* ≤ 1.69 × 10^–10^) (Table 1, Fig. 2).

**Table 1:** Mendelian randomization analyses causal relationships between MS and psoriasis using genetic variations, and vice versa.

Exposure	Outcome	Method	OR	95% CI	*p*
MS	Psoriasis	CAUSE	0.93	(0.87, 1.00)	0.045
		IVW	0.93	(0.91, 0.94)	2.51 × 10^–20^
		Weighted median	0.92	(0.89, 0.94)	1.52 × 10^–13^
		RAPS	0.92	(0.91, 0.94)	< 1.00 × 10^–16^
		IVW radial	0.92	(0.91, 0.94)	1.76 × 10^–20^
		MR-PRESSO	0.92	(0.91, 0.94)	4.12 × 10^–14^
Psoriasis	MS	CAUSE	0.72	(0.53, 0.97)	0.001
		IVW	0.71	(0.67, 0.76)	4.80 × 10^–26^
		Weighted median	0.67	(0.62, 0.73)	2.06 × 10^–18^
		RAPS	0.69	(0.65, 0.74)	< 1.00 × 10^–16^
		IVW radial	0.70	(0.66, 0.74)	4.96 × 10^–33^
		MR-PRESSO	0.70	(0.66, 0.74)	4.17 × 10^–12^

CI, confidence interval; CAUSE, causal analysis using summary effect estimates; IVW, inverse variance-weighted; MS, multiple sclerosis; OR, odds ratio; MR-PRESSO, MR Pleiotropy RESidual Sum and Outlier; RAPS, robust adjusted profile score.

**Figure 3:**
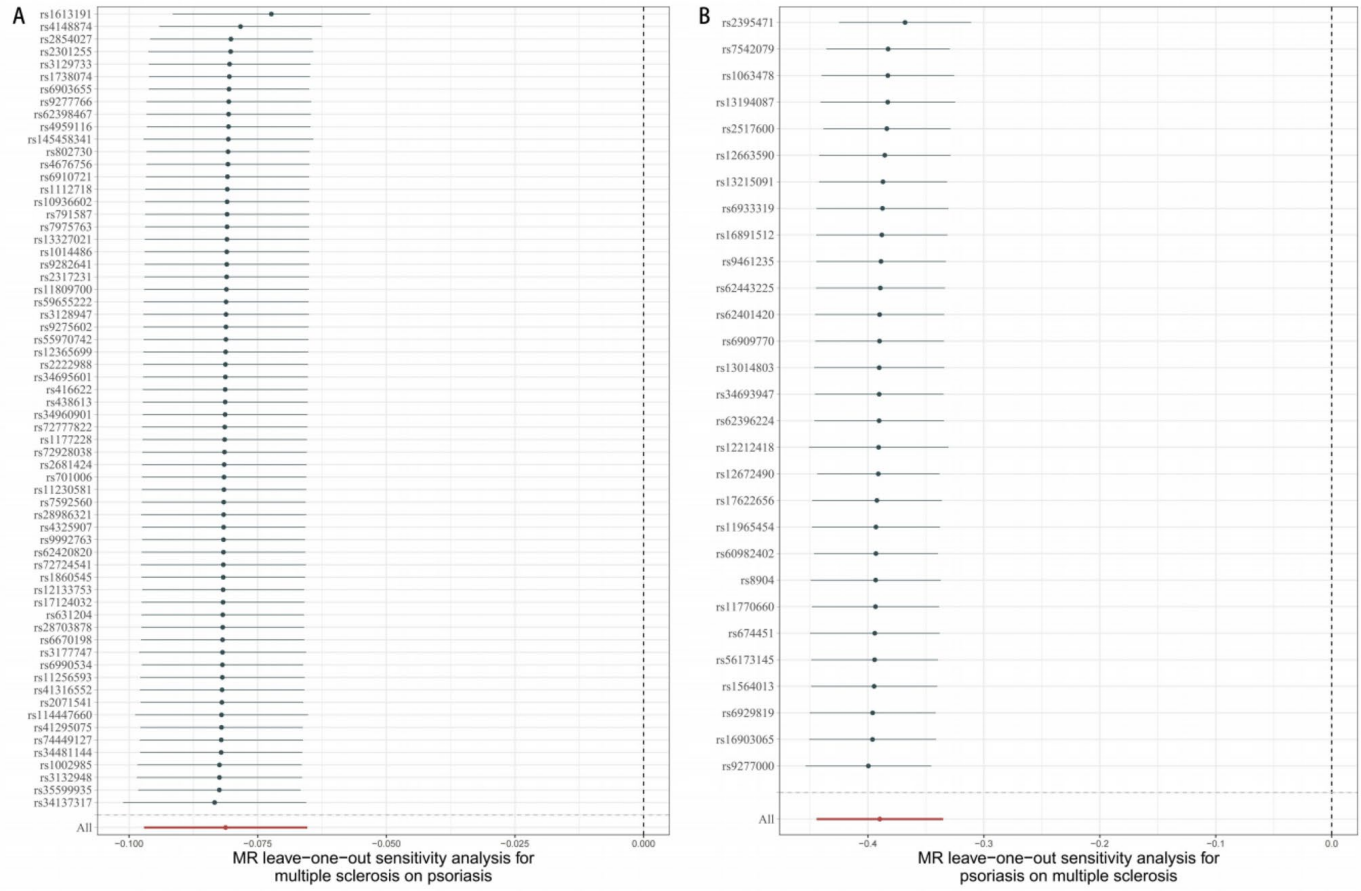
(**A**) The leave-one-out plot of SNPs associated with multiple sclerosis and their risk on psoriasis; (**B**) The leave-one-out plot of SNPs associated with psoriasis and their risk on multiple sclerosis. SNPs, single nucleotide polymorphisms.

The results showed no significant heterogeneity and pleiotropy among the selected genetic instruments (Table 2), and no SNPs with high influence were found by leave-one-SNP-out analysis (Fig. 3). Low or no heterogeneity and the same results from the pleiotropy-robust methods, implicitly ensured the independence and exclusion limitation assumptions in the MR.

**Table 2:** Heterogeneity of Wald ratios and MR-Egger test for directional pleiotropy.

Exposure	Heterogeneity			
	Outcome	Q	*df*	*p*-value
Psoriasis	MS	28.140	27	0.404
MS	Psoriasis	74.147	63	0.139

Exposure	MR-Egger test for directional pleiotropy			
	Outcome	Intercept	SE	*p*-value
Psoriasis	MS	0.021	0.011	0.055
MS	Psoriasis	0.003	0.003	0.359

df, degree of freedom; MS, multiple sclerosis; MR, Mendelian randomization; Q, heterogeneity statistic Q.

### Genetic correlation between MS and psoriasis

The total heritability of psoriasis was 1.97%, 95%CredIn: 1.32–2.26% (mean χ^2^ = 1.19). The total heritability of MS was 9.00%, 95%CredIn: 7.31–10.69% (mean χ^2^ = 1.23). The LDSC did not support genetic correlation between psoriasis and MS (rg = − 0.071, *p* = 0.2852).

## Discussion

We used bidirectional MR to investigate the causal association between MS and psoriasis. Overall, there was no significant proof that MS increases the risk of psoriasis and vice versa, and conversely, we found that the genetic liability to MS may decrease the risk of psoriasis and vice versa.

The MR analysis, especially CAUSE—which is designed to detect the possible small causal association between MS and psoriasis—utilized the full range of genetic variations and did not provide genetic evidence for an association between MS and psoriasis, and other complementary MR methods reached the same conclusion as CAUSE. Our findings contradict previous meta-analyses, which have suggested a bidirectional positive association between MS and psoriasis^18,20^. Although pathogenic links between MS and psoriasis have been proposed due to their shared immune response pathways^42–44^, our MR study findings did not show evidence of such a positive correlation as a two-way causal link. One possibility could be that unidentified confounding factors interfere with the previously reported relationship between MS and psoriasis. In addition, observational studies cannot determine the causal connection between MS and psoriasis since the majority of individuals with psoriasis display systemic health issues. Psoriasis is frequently accompanied by a wide variety of systemic disorders, such as disorders of the cardiovascular system, joints, and central nervous system^5^, and both MS and psoriasis have numerous comorbidities^6,7^. Thus, the reported positive association between MS and psoriasis may be attributable to these comorbidities and shared inflammatory pathways.

Most prior research on the correlation between psoriasis and MS has been limited by small sample sizes and primarily observational in nature^45^. Observational studies investigating the relationship between MS and psoriasis have yielded conflicting findings, with some studies shown a bidirectional positive association between MS and psoriasis^18,20^, but others shown no association^46^. To our knowledge, this is the first MR study that focused on the causal association between MS and psoriasis, and found that the genetic liability to MS may decrease the risk of psoriasis and vice versa. Although similar have not been reported in previous observational studies, the immunological mechanism linking elevated IL-27 in psoriasis^47,48^ and decreased IL-27 in MS^49,50^ may shed light on this association. In addition, it has been found that the immunological effects of low levels of IL-27 in MS patients with psoriasis comorbidity have been suggested to be mitigated by the reverse impact of high levels of IL-27 generated by psoriasis^51^. Furthermore, there had been several reports of psoriasis occurring during MS treatment^52–54^ as well as MS occurring during psoriasis treatment^55–57^. All these findings suggest that MS may reduce the risk of psoriasis and vice versa.

Our research has several advantages. First, using MR can reduce bias caused by unknown reverse causality and confounding factors and apply to causal reasoning, which can be impossible to achieve in traditional observational studies. Second, the CAUSE and IVW methods produced the same results, and the sensitivity analysis also reached the same conclusion, which improved the robustness and credibility of the conclusion. Third, reliable analytical data for this study came from the large psoriasis GWAS dataset (N = 252,323 individuals of European ancestry) and the MS GWAS dataset (N = 115,803 individuals of European ancestry). Importantly, LDSC further revealed no genetic correlation between MS and psoriasis. Fourth, the GWAS datasets for both MS and psoriasis were collected from people of European descent, which may have lessened the impact of demographic stratification on potential associations. Fifth, we carried out a pleiotropic investigation to examine the possibility of genetic variations being linked to recognized risk factors for MS and psoriasis. We ruled out three genetic variations linked to potential confounding factors in psoriasis and two in MS since doing so was compatible with the MR assumption.

Nevertheless, our MR study does contain a few restrictions that cannot be disregarded and should be considered carefully. First, MR based on genetic summary statistics narrowed down the scope of the analysis. However, the observed consistency across multiple analytical approaches makes it highly unlikely that this outcome was influenced by bias. Second, the existence of potentially confounding factors is not something that can be fully eliminated. The biggest problem during MR studies is pleiotropy, and horizontal pleiotropy emerges when genetic variation influences results through multiple pathways. Although we identified pleiotropy using the most advanced methods available, such as CAUSE^34^, it has been nearly impossible to entirely exclude the presence of pleiotropy in MR studies thus far^58–60^. Third, there may be racial variations in the genetic link between MS and psoriasis, whereas our GWAS dataset was derived from European populations and may not be effectively extended to other populations. It is possible for hidden population structure to interfere with the connection between genetic variation and outcome. Fourth, MR analysis only allows for the preliminary judgment of the causality between MS and psoriasis, and further investigation into the mechanism of the negative causal relationship between MS and psoriasis is needed. Last but not least, in the pyramid of evidence-based medicine, MR studies lie between RCT and observational studies^61^, so the causal link between MS and psoriasis needs to be further investigated in RCT.

## Conclusion

Overall, the results of our study supplied genetic evidence suggesting that the genetic liability to MS reduces the risk of psoriasis and vice versa. The LDSC showed no genetic correlation between psoriasis and MS. Our investigations may have implications for the management of co-occurring MS and psoriasis.

### Supplementary Information

Supplementary Tables.

## Data Availability

https://github.com/alidang1/MR-CODE.
