# Supplementary material for: Reverse causation between multiple sclerosis and psoriasis: a genetic correlation and Mendelian randomization study
Source: Sci Rep. 2024 Apr 17;14:8845. doi: 10.1038/s41598-024-58182-9 (PMC11024188; doi:10.1038/s41598-024-58182-9)
Supplement: Supplementary file 1 — Supplementary Tables. [file 41598_2024_58182_MOESM1_ESM.docx]

**Table S1.** **SNPs used as instruments for multiple sclerosis at the genome-wide level of significance.**

| SNP | Alt | Ref | Beta | SE | *P* | F |
| --- | --- | --- | --- | --- | --- | --- |
| rs1002985 | T | C | 0.6994 | 0.0346 | 1.22E-90 | 407.5932 |
| rs1014486 | C | T | 0.1051 | 0.0164 | 1.36E-10 | 41.2144 |
| rs10936602 | C | T | -0.1084 | 0.0192 | 1.68E-08 | 31.8368 |
| rs11230581 | T | C | 0.1288 | 0.0180 | 8.16E-13 | 51.2436 |
| rs11256593 | T | C | 0.1863 | 0.0174 | 6.78E-27 | 115.2954 |
| rs114447660 | A | G | -1.6977 | 0.1317 | 5.34E-38 | 166.0702 |
| rs1177228 | G | A | 0.1074 | 0.0187 | 8.57E-09 | 33.1415 |
| rs11809700 | T | C | 0.1444 | 0.0184 | 3.51E-15 | 61.9602 |
| rs12133753 | T | C | -0.1248 | 0.0222 | 1.92E-08 | 31.5779 |
| rs12365699 | A | G | -0.1438 | 0.0228 | 3.15E-10 | 39.5817 |
| rs13327021 | T | C | 0.1152 | 0.0171 | 1.73E-11 | 45.2580 |
| rs145458341 | T | G | -2.3037 | 0.1685 | 1.55E-42 | 186.8517 |
| rs1613191 | A | G | 2.0356 | 0.1432 | 7.07E-46 | 202.1573 |
| rs17124032 | A | G | -0.2168 | 0.0316 | 7.08E-12 | 47.0047 |
| rs1738074 | C | T | 0.1137 | 0.0167 | 9.91E-12 | 46.3466 |
| rs1860545 | A | G | 0.1165 | 0.0170 | 7.79E-12 | 46.8188 |
| rs2071541 | G | A | -0.1852 | 0.0257 | 5.24E-13 | 52.1125 |
| rs2222988 | G | A | -0.2434 | 0.0405 | 1.81E-09 | 36.1726 |
| rs2301255 | A | G | -0.4362 | 0.0244 | 9.50E-72 | 320.8420 |
| rs2317231 | T | G | -0.1006 | 0.0167 | 1.90E-09 | 36.0714 |
| rs2681424 | C | T | -0.1212 | 0.0166 | 2.71E-13 | 53.4101 |
| rs2854027 | C | T | -0.2289 | 0.0336 | 9.40E-12 | 46.4499 |
| rs28703878 | G | A | 0.1336 | 0.0214 | 4.51E-10 | 38.8800 |
| rs28986321 | A | C | -0.9446 | 0.1058 | 4.26E-19 | 79.7469 |
| rs3128947 | G | A | -0.2945 | 0.0343 | 8.33E-18 | 73.8738 |
| rs3129733 | G | A | -0.2845 | 0.0255 | 6.54E-29 | 124.5013 |
| rs3132948 | G | T | -0.3375 | 0.0175 | 4.62E-83 | 372.7915 |
| rs3177747 | A | G | -1.1849 | 0.1379 | 8.42E-18 | 73.8514 |
| rs34137317 | T | C | 4.9063 | 0.5214 | 4.93E-21 | 88.5593 |
| rs34481144 | T | C | 0.1577 | 0.0282 | 2.34E-08 | 31.1871 |
| rs34695601 | C | T | -0.1095 | 0.0198 | 3.16E-08 | 30.6036 |
| rs34960901 | T | C | 0.1425 | 0.0242 | 3.96E-09 | 34.6445 |
| rs35599935 | C | T | -0.5099 | 0.0448 | 5.55E-30 | 129.3973 |
| rs41295075 | T | C | -0.2942 | 0.0334 | 1.34E-18 | 77.4859 |
| rs41316552 | C | T | -0.2954 | 0.0251 | 7.35E-32 | 137.9837 |
| rs4148874 | T | C | -0.4858 | 0.0185 | 2.25E-151 | 686.7726 |
| rs416622 | C | T | -0.1050 | 0.0169 | 5.80E-10 | 38.3868 |
| rs4325907 | T | C | -0.0993 | 0.0168 | 3.68E-09 | 34.7851 |
| rs438613 | C | T | 0.1380 | 0.0166 | 9.43E-17 | 69.0838 |
| rs4959116 | T | C | -0.2001 | 0.0240 | 6.63E-17 | 69.7806 |
| rs55970742 | T | C | -0.1004 | 0.0179 | 2.05E-08 | 31.4468 |
| rs59655222 | C | T | -0.1232 | 0.0186 | 3.76E-11 | 43.7360 |
| rs62398467 | T | C | -0.1993 | 0.0201 | 3.48E-23 | 98.3678 |
| rs62420820 | A | G | 0.1372 | 0.0188 | 2.50E-13 | 53.5694 |
| rs631204 | A | C | 0.1128 | 0.0164 | 6.45E-12 | 47.1882 |
| rs6670198 | C | T | -0.1450 | 0.0176 | 2.03E-16 | 67.5743 |
| rs6903655 | T | C | 0.1207 | 0.0207 | 5.81E-09 | 33.8960 |
| rs6910721 | G | A | -0.1554 | 0.0273 | 1.28E-08 | 32.3599 |
| rs6990534 | G | A | 0.1071 | 0.0182 | 3.60E-09 | 34.8304 |
| rs701006 | G | A | 0.1139 | 0.0168 | 1.35E-11 | 45.7415 |
| rs72724541 | A | G | 1.0933 | 0.1564 | 2.74E-12 | 48.8698 |
| rs72777822 | A | C | -0.7586 | 0.1390 | 4.85E-08 | 29.7749 |
| rs72928038 | A | G | 0.1605 | 0.0248 | 9.01E-11 | 42.0255 |
| rs74449127 | G | A | -0.1970 | 0.0256 | 1.36E-14 | 59.2897 |
| rs7592560 | A | G | 0.1041 | 0.0165 | 2.87E-10 | 39.7584 |
| rs791587 | G | A | -0.0979 | 0.0162 | 1.45E-09 | 36.5964 |
| rs7975763 | T | C | 0.1210 | 0.0210 | 7.80E-09 | 33.3231 |
| rs802730 | C | T | -0.1143 | 0.0182 | 3.24E-10 | 39.5266 |
| rs9277766 | T | C | -0.2824 | 0.0236 | 7.34E-33 | 142.5599 |
| rs9282641 | A | G | -0.2091 | 0.0306 | 8.39E-12 | 46.6725 |
| rs9992763 | T | G | -0.0900 | 0.0165 | 4.51E-08 | 29.9151 |

**Table S2. SNPs used as instruments for psoriasis at the genome-wide level of significance.**

| SNP | Alt | Ref | Beta | SE | *P* | F |
| --- | --- | --- | --- | --- | --- | --- |
| rs1063478 | T | C | 0.2894 | 0.0244 | 2.39E-32 | 140.2145 |
| rs11770660 | T | C | 0.1345 | 0.0216 | 4.99E-10 | 38.6798 |
| rs11965454 | C | T | -0.2455 | 0.0374 | 5.43E-11 | 43.0147 |
| rs12212418 | T | C | 0.2777 | 0.0163 | 4.50E-65 | 290.1972 |
| rs12672490 | T | C | 0.1624 | 0.0262 | 5.81E-10 | 38.3851 |
| rs13014803 | C | T | -0.1154 | 0.0199 | 6.49E-09 | 33.6830 |
| rs13194087 | A | G | 0.5531 | 0.0316 | 1.65E-68 | 305.9662 |
| rs13215091 | A | G | 0.1848 | 0.0312 | 3.14E-09 | 35.0978 |
| rs1564013 | G | A | -0.0938 | 0.0166 | 1.77E-08 | 31.7335 |
| rs16891512 | A | G | 0.3794 | 0.0354 | 7.74E-27 | 115.0341 |
| rs16903065 | A | C | -0.1577 | 0.0260 | 1.27E-09 | 36.8644 |
| rs17622656 | A | G | 0.0986 | 0.0180 | 4.14E-08 | 30.0827 |
| rs2517600 | A | G | -0.1600 | 0.0181 | 8.69E-19 | 78.3367 |
| rs34693947 | A | G | 0.1769 | 0.0262 | 1.35E-11 | 45.7463 |
| rs56173145 | A | G | 0.1254 | 0.0215 | 5.59E-09 | 33.9718 |
| rs60982402 | T | G | 0.2077 | 0.0371 | 2.14E-08 | 31.3662 |
| rs62396224 | G | A | 0.2334 | 0.0301 | 8.79E-15 | 60.1499 |
| rs62401420 | C | T | 0.4090 | 0.0335 | 2.49E-34 | 149.2824 |
| rs62443225 | A | G | 0.2061 | 0.0286 | 5.89E-13 | 51.8827 |
| rs674451 | C | T | 0.1255 | 0.0166 | 3.89E-14 | 57.2256 |
| rs6909770 | T | C | 0.3880 | 0.0531 | 2.63E-13 | 53.4642 |
| rs6929819 | G | A | 0.1001 | 0.0159 | 3.13E-10 | 39.5912 |
| rs6933319 | T | C | 0.3763 | 0.0366 | 7.42E-25 | 105.9869 |
| rs7542079 | C | T | 0.0950 | 0.0162 | 4.06E-09 | 34.5941 |
| rs8904 | A | G | -0.1173 | 0.0165 | 1.19E-12 | 50.5016 |
| rs9277000 | T | C | 0.2175 | 0.0254 | 9.48E-18 | 73.6166 |
| rs9461235 | C | T | -0.1308 | 0.0209 | 4.12E-10 | 39.0533 |
